# First report of bovine viral diarrhea virus subgenotypes 1d and 1e in southern Chile

**DOI:** 10.1186/s12985-023-02170-4

**Published:** 2023-09-07

**Authors:** Florence Hugues, Ignacio Cabezas, Mutien Garigliany, Felipe Rivas, Tomás Casanova, Eddy E. González, Oliberto Sánchez, Raúl Castillo, Natalie C. Parra, Oscar Inostroza-Michael, Lucila Moreno, Cristián E. Hernández, Jorge R. Toledo

**Affiliations:** 1 Departamento de Ciencias Clínicas, Facultad de Ciencias Veterinarias, Universidad de Concepción, Chillán, Chile; 2 Department of Pathology, Faculty of Veterinary Medicine, Université de Liège, Liège, Belgium; 3 Laboratorio de Biotecnología Y Biofármacos, Departamento de Fisiopatología, Facultad de Ciencias Biológicas, Universidad de Concepción, Víctor Lamas 1290, 4070386 Concepción, Chile; 4 Vetagro Patagonia, Coyhaique, Chile; 5 Laboratorio de Ecología Evolutiva y Filoinformática, Departamento de Zoología, Facultad de Ciencias Naturales y Oceanográficas, Universidad de Concepción, Concepción, Chile; 6 Centro de Investigación en Recursos Naturales, HOLON SpA., Concepción, Chile; 7 Laboratorio de Ecología Parasitaria, Departamento de Zoología, Facultad de Ciencias Naturales y Oceanográficas, Universidad de Concepción, Concepción, Chile; 8 Universidad Católica de Santa María, Arequipa, Perú

**Keywords:** Bovine viral diarrhea virus, Genetic diversity, Phylogenetic analysis

## Abstract

Bovine viral diarrhea virus (BVDV) affects cattle worldwide causing severe productive and economic loss. In this study, we investigated the subgenotypes of BVDV circulating in cattle samples from the Aysén region, an active cattle breeding area located in southern Chile. Partial amplification of the 5' untranslated region (UTR) was performed by polymerase chain reaction (PCR), and twelve samples were analyzed by Sanger sequencing and phylogenetic analysis. Eight samples were identified as belonging to *Pestivirus bovis* subgenotype 1e, three to 1-b, and one to 1-d. The phylogenetic analyses performed revealed a marked distance between these now-identified strains and those previously reported in the country. These findings support the need to continually expand the analysis of the variability of the viral phylogeny for the currently circulating BVDV strains and to update the vaccines recommended for this livestock area and surrounding areas.

**Supplementary Information:**

The online version contains supplementary material available at 10.1186/s12985-023-02170-4.

## Introduction

Bovine viral diarrhea virus (BVDV) is an infectious agent of worldwide distribution, causing serious economic losses associated with reproductive disorders, low milk production, calf growth retardation, congenital defects, respiratory and digestive disorders, among others [3, 12, 21]. Therefore, the implementation of viral control programs is necessary and has an appreciable impact on the optimization of production costs in animal husbandry [23]. Persistently infected (PI) calves are born with a transplacental infection and are the main source of viral spread, as they continuously disseminate BVDV through all body secretions in the herd. [4, 16].

BVDV belongs to the family *Flaviviridae*, genus *Pestivirus*, within which four principal species are recognized: *Pestivirus bovis* (BVDV-1 or *Pestivirus A*), *Pestivirus tauri* (BVDV-2 o *Pestivirus B*), *Pestivirus suis (Classical swine fever* virus (CSFV) o *Pestivirus C*) and *Pestivirus ovis (*or *Border disease* virus (BDV) *Pestivirus D*) [13, 32]. The BVDV genome is a positive single stranded RNA with a large open reading frame coding for a single polyprotein of 3900 amino acids, which is post translationally cleaved [32]. Phylogenetic studies on BVDV are based on the analysis of diverse regions of the genome, most frequently the 5’UTR, which is the most conserved, followed by the coding sequences of the N^pro^ and E2 proteins [25, 35]. Worldwide, 21 subgenotypes of *Pestivirus bovis* and 4 subgenotypes for *Pestivirus tauri* have been reported so far, with subgenotypes 1a and 1b being the most represented [35]. However, additional subgenotypes were proposed recently [5, 27]. In Chile, besides 1a and 1b, subgenotypes 1c, 1j and undefined *Pestivirus tauri* have been reported for central and central-south regions of the country in studies performed fifteen years ago [7, 24]. Nevertheless, considering the very rapid rate of RNA virus evolution, the BVDV could produce a plethora of genomic variants within a single host or in a population in a short time [6, 10]. Continuous monitoring of BVDV is very necessary, given its relevance for decision making regarding the implementation of vaccination and infection mitigation programs, and its continued spread and dispersal. In Chile, the Aysén cattle region is of particular interest, since 94% of the breeders only keep calves until they are 8–10 months old [11]. Subsequently, the animals are moved to northern regions to finish fattening. In addition, this is a region of great importance in the supply of breeding males and females to other regions of the country. All this mobility of animals also contributes to the dispersion of different strains of BVDV, not only within Chile, but also with neighboring countries such as Argentina.

The main objective of this study was to determine the possible variability of BVDV subgenotypes present in cattle in the Aysén region, in southern Chile, and their possible origin based on phylogenetic relationships between the currently circulating strains and those previously reported in other regions of the country.

## Methods

Serum samples were collected from apparently healthy bovine cattle hold in a quarantine property in the region of Aysén, before exportation in 2017. The 1331 sampled animals, males and females, black and red Angus breed, ranged from 7 to 20 months old, and came from different locations within the four provinces of the region. On the arrival of animals from a particular property to the quarantine field, they were kept separated from the other animals until the sampling was done and the animals were confirmed to be negative, to avoid infection between different groups. All animals were tested with an antigenic ELISA test (ELISA IDEXX BVDV ag/Serum Plus™). RNA was extracted from the positive samples using Trizol LS® (Invitrogen, USA) according to manufacturer’s specifications. The complementary DNA was synthesized with RevertAid First Strand cDNA Synthesis kit (Thermo Scientific™) under the following conditions: 5 min at 25 °C, 60 min at 42 °C and 5 min at 70 °C. From the obtained cDNA, sequences of the 5’-UTR (288 base pairs) were amplified by PCR using the primers 324/326 [33]: forward 5’-ATGCCCTAGTAGGACTAGCA-3’ and reverse 5’-TCAACTCCATGTGCCATGTAC-3’. The amplification was performed using Platinum SuperFI II PCR Master Mix (Invitrogen™), with 10 µl of sample in a final volume of 50 µl, beginning with 1 min at 94 °C and followed by 45 cycles programmed as: 5 s at 98 °C, 10 s at 55 °C and 15 s at 72 °C; and a final extension of 3 min at 72 °C. The amplified products were separated in 1,3% agarose gel ran at 110 V. The sequencing of the amplicons was carried out in the Pathology Department, Faculty of Veterinary Medicine, Université de Liège, Belgium, using the Sanger Method. For typing, the results were submitted to a sequence comparison analysis with the Basic Local Alignment Search Tool (BLAST®) software from the US National Center for Biotechnology Information (https://blast.ncbi.nml.nih.gov/Blast.cgi).

The phylogenetic analysis was based on Bayesian Inference with Markov Chain Monte Carlo algorithm (BMCMC) as implemented in BayesPhylogenies v1.1 software (http://www.evolution.rdg.ac.uk/BayesPhy.html). This model use a general likelihood-based mixed model (MM) of gene sequence evolution as described by Pagel and Meade [19, 20], allowing the accommodation of cases in which different sites in the alignment evolved in qualitatively distinct ways, but does not require prior knowledge about these alignment patterns or partitioning of the data. Two independent analyses of 15 million iterations were ran, sampling every 1000 iterations and 10% of iterations were discarded from each analysis to avoid the inclusion of phylogenetic trees outside the convergence zone. Next, both analyses were mixed and from this final sample, a consensus tree was built using the Tree Annotator v2.5.2 software (https://www.beast2.org/treeannotator/).

To produce rooted phylogenetic trees, sequences of *Pestivirus tauri* were selected as external group (GenBank accession numbers: U18059, AY671984, AY671985, AY671986, AY671987 and AF356505). To delimit the existence of discrete genetic units on a rooted phylogenetic tree, we used a model of speciation or branching events in terms of the number of substitutions in the “multi-rate Poisson Tree Process” (mPTP) software [15]. The analyses were performed under Bayesian inference, executing four chains of Markov Monte Carlo with 50 million iterations, and sampling every 10,000 iterations. Then, the four chains were mixed. Complementarily, we visualize the genetic variation patterns of BVDV genetic diversity by constructing a haplotype network using the median joining method proposed by Bandelt et al. [2] implemented in the NETWORK software (http://www.fluxus-engineering.com/sharenet).

The identity percentage of the nucleotide sequences from the 5’UTR region was calculated with the Sequence Demarcation Tool (SDT v1.2) software [18], based on the algorithms of the Multiple Sequence Comparison by Log-Expectation (MUSCLE) [8]. The sequences of strains previously reported in Chile were downloaded from NCBI data base, using the GenBank accession numbers published in Pizarro-Lucero et al. [24] and in Donoso et al. [7], as well as reference sequences of genotypes 1a (M31182 y AF298061), 1b (M96687 y AF298070), 1c (AF049221 y JQ743606), 1d (AF298065 y AF298065), 1e (AF298062 y AF298054), 1f (AF298067), 1g (AF298064), 1h (AF298066), 1i (AF298059), 1j (AB078950 y U97429), 1k (AF117699), 1l (KF205306), 1m (AF526381), 1n (DQ973181 y GQ495676), 1o (AB300691), 1p (GU120248), 1q (JN248727), 1r (LM994672), 1s (LM994673), 1t (LM994674), 1v (MN442377) y 1w (MN417892).

## Results and discussion

**Fig. 1:**
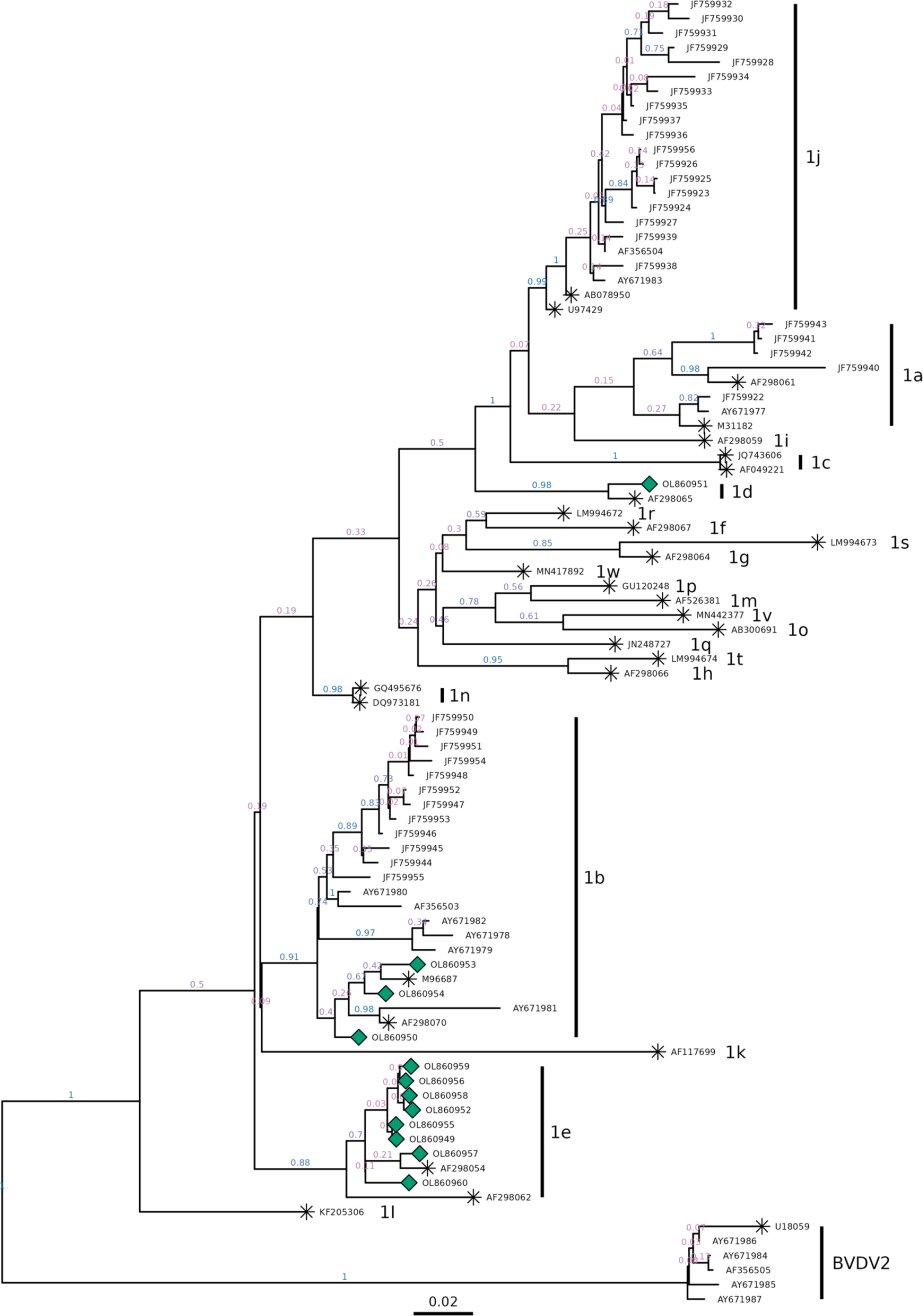
Bayesian consensus phylogenetic tree based on the 5'UTR sequences of Chilean isolates (ref [6] and [21], without symbol), references strains (identified with *) and the 12 strains under study from the Aysén region (green diamond). Values behind the nodes indicate posterior probability values, with a colour gradient between low (0.0) in red to high (1.0) in blue. Subgenotypes are indicated in the right side

From the 24 antigenically positive serum samples, 12 amplicons were obtained for sequencing procedures. The low recovery of amplicons is probably due to RNA degradation consecutive to the delay between the first analysis of the samples and the reception of them in our laboratories, what led to repeated frozen-thaw cycles [28]. According to the BLAST analysis of the obtained sequences, all the samples belonged to *Pestivirus bovis*, from which eight corresponded to subgenotype 1e, three samples were classified as subgenotype 1b and one as 1d (Table 1). The phylogenetic tree confirmed the species classification of the samples, as well as their subgenotypes (Fig. 1). From the resulting consensus tree, eight samples were classified as clade 1e by 0.70 of posterior probability (pp) and forming a separate cluster from the strains earlier reported in Chile (Fig. 1, pp = 0.89). The distance with the closest clade, which corresponds to sequences of subgenotypes 1b (pp = 0.92), showed 0.062 changes per nucleotide.

**Table 1:** General information about the BVDV field isolates analyzed in this study. The samples were collected on june 2017

Serum Sample id	Age at sample time (months)	Animal origin (Within Aysén Region)	Subgenotype	GenBank accession numbers
COY1-17	9	Coyhaique	1e	OL860949
COY2-17	8	Aysén	1b	OL860950
COY5-17	8	Coyhaique	1d	OL860951
COY7-17	13	Coyhaique	1e	OL860952
COY10-17	8	Coyhaique	1b	OL860953
COY11-17	15	Coyhaique	1b	OL860954
COY13-17	8	Chile Chico	1e	OL860955
COY14-17	8	Aysén	1e	OL860956
COY16-17	20	Coyhaique	1e	OL860957
COY19-17	8	Cisnes	1e	OL860958
COY21-17	8	Río Ibáñez	1e	OL860959
COY24-17	9	Aysén	1e	OL860960

Three of our samples from Aysén (i.e., OL860953, OL860954 and OL860950) belonged to clade 1b. These samples cluster to a subclade together with M96687 from Osloss (Germany), AF298070 from Austria and AY671981 from Central Chile (pp = 0.92). The BLAST analysis revealed the highest similarity with strains reported from Argentina, United Kingdom and Italy (Additional file 2: Table S1). The strain from Argentina was recently registered and corresponds to a sample taken in 2019, posterior to our sampling date. These results seem to indicate that 1b strains may have a European origin, but it is difficult to explain the route of transmission, as importation of live animals is not a common practice for Chile, and semen used for artificial insemination comes mainly from U.S.A. and Canada and must be certified free of BVDV and other pathogens. Only one of analyzed samples (i.e., OL860951) belongs to the clade 1d and has over 97% identity with strains from Brazil and China (Additional file 2: Table S1). The Brazilian strain corresponds to a recent sampling while the Chinese strain was collected ten years ago.

**Fig. 2:**
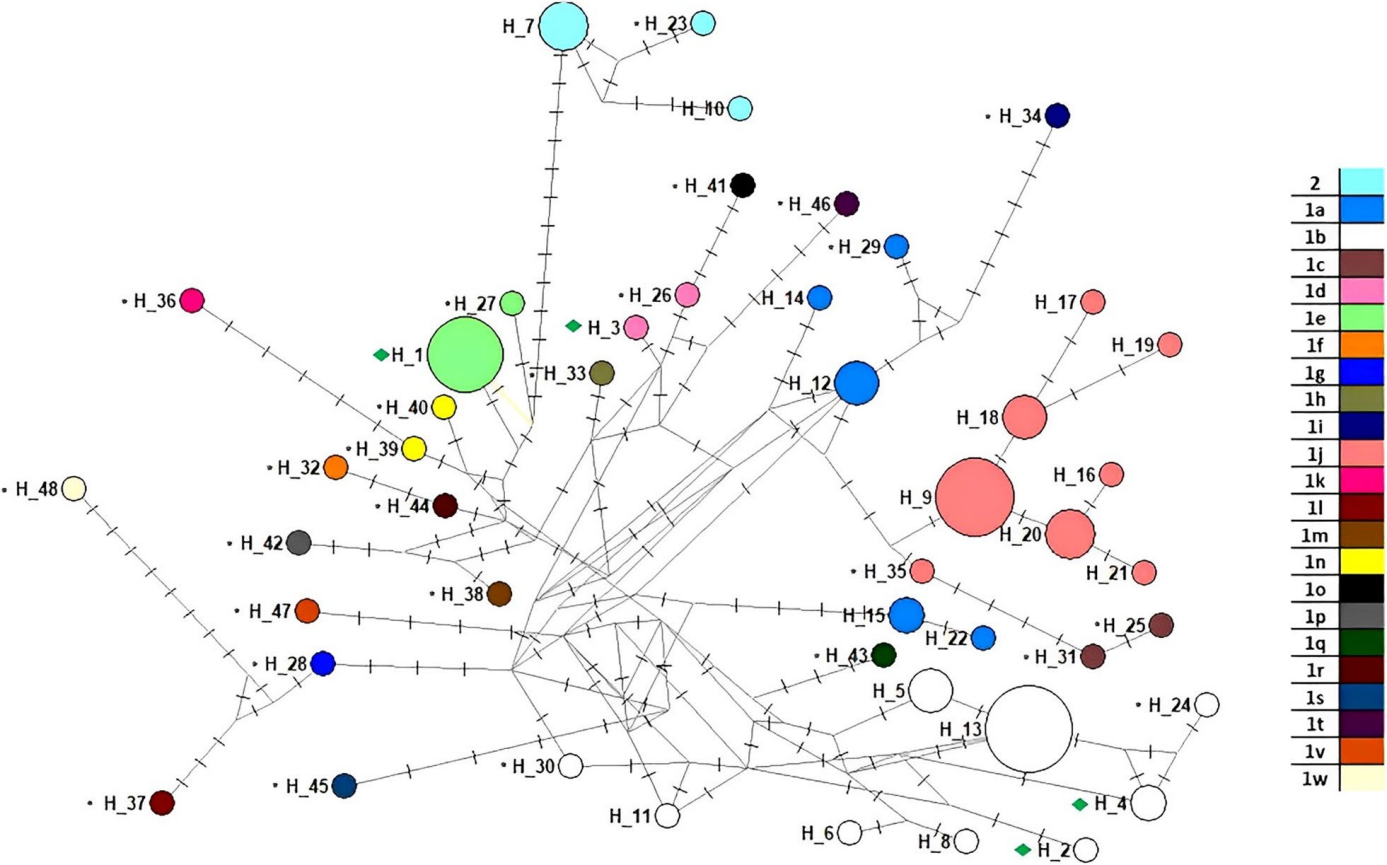
Median-joining haplotype network tree based on the 5'UTR sequences of Chilean isolates (ref [6] and [21], without symbol), references strains (identified with*) and of the 12 strains under study from the Aysén region (green diamond). Haplotypes are identified by the numbers in the circles; circle’s size is proportional to its frequency. Hatches on the lines represent mutational steps. Colors within the circles represent the strains where haplotypes were found

The existence of discrete genetic units based on mPTP analysis confirms the units 1a, 1b, 1d, 1e, 1j and *Pestivirus tauri*. Strains belonging to subgenotypes 1f, 1g, 1h, 1m, 1o, 1p, 1q, 1r, 1s, 1t, 1v and 1w form a single unit (Additional file 1: Fig. S1). Genetic diversity analyzed using a haplotype network based on haplogroups highlights the presence of one haplotype for 1e unit, gathering our 8 samples from Aysén together with AF298054, and well-defined units based on differentiated haplotypes proximity (Fig. 2). SDT pair-wise ranking of the sequences showed an identity between 99.1 and 100% for samples 1e; 94.8 to 98.8% for samples 1b; while the sample 1d had a mean identity of 87.5% and 87.4% with samples 1e and 1b, respectively. As for the comparison between samples 1e and 1b, the identity ranged from 89.0 to 90.5%.

Genotyping the 5’UTR is commonly used as it is a highly conserved region for BVDV [1, 34]. The sequencing of the isolates from the Aysén region allowed reporting BVDV subgenotypes 1e and 1d for the first time in Chile. Previously, based on 5'UTR sequencing, subgenotypes 1a, 1b, 1c and *Pestivirus tauri* had been reported in sampled animals from the central regions of Chile between 1993 and 2001[24]. Later, in samples taken between years 2003 and 2007 from central and south-central regions of the country, the presence of subgenotypes 1a and 1b were confirmed, adding subgenotype 1j, based on the sequencing of 5’UTR and E2 genomic sequences [7]. Reports from Argentina only showed the presence of subgenotypes 1a, 1b and *Pestivirus tauri* [14, 22], even though recently, subgenotypes 1e and 1i were reported but with only n = 1 for each strain [31]. The major risk of contact with animals from this neighbor country may occur because of the use of summer fields located along the border. On the continent, only in Brazil were reported strains from subgenotypes 1d and 1e previously [29, 35], and there are not movements of cattle registered between Brazil and Chile, though, it is unlikely that a transmission occurs through this way. But recently, a new 1e strain from Brazil was registered in GenBank data base and it has between 99.56 and 100% identity with the 1e strains from Aysén (Additional file 2: Table S1). The 1e strains under study also have high identity (between 99.17 and 100%) with strains from United Kingdom, Switzerland, and Italy (Supplementary Table S2), which indicates again the possibility of a European origin. Besides, in our evaluation, subgenotype 1b was found, in agreement with previous reports in the country [7, 24]. This result was expected, since subgenotype 1b is the most represented subgenotype worldwide [35], even though, in this case, a higher frequency (66.7%) was observed for subgenotype 1e versus 25% for 1b.

In Chile, even if there are some producers who vaccinate their cattle against BVDV, the percentage of effectively vaccinated animals is unknown. The only commercial vaccine available in the country is inactivated and contain strains 1a and 2 (Cattle Master Gold FP5®) and until two years ago, also another vaccine containing strains 1a and 1b (Cattle Master®). In a study carried out in the USA, it was suggested that the regression over time of one subgenotype in a particular region might be related to the use of vaccine strains targeting the same subgenotype, which would induce a change in dominance of the current subgenotypes [26]. This fact could partly explain the lower frequency of subgenotype 1b in the samples studied, although a larger number of samples as well as comparison with samples from the region in previous years is required to strongly corroborate this explanation. Moreover, studies have shown that different vaccines, including killed and live attenuated vaccines, composed with strains 1a or 1b, have varying levels of neutralizing antibodies against heterologous subgenotypes of 1a or 1b strains [9, 30]. Additionally, these vaccines have been found to have a total absence of neutralizing antibodies against 1e strains [30]. This highlights the importance of incorporating circulating strains in the formulation of new BVDV vaccines to promote multivalent protection.

Phylogenetic trees inferred from single gene sequences are gene trees rather than species trees, although hierarchical relationships are expected to be consistent with species trees [36]. In this case, the phylogenetic tree obtained represents the intraspecific variations of the sequences analyzed. Network analysis is a tool used to establish and visualize the number of sites that are different between two sequences. For closely related sequences, this represents a good estimation of the number of mutations separating both sequences from their common ancestors [17]. Here, the diversity of strains found, and the high number of mutations observed are indicators of the existence of a pathway of virus introduction related with another genetic origin that was not present in the region of Aysén. Due to the low number of samples analyzed here, it is necessary to integrate more data, like the sequences of multiple genes, to validate the relationships [36].

The present results, added to previous studies, reveal a great diversity of BVDV subgenotypes circulating in Chile. In addition, it provides preliminary evidence of the variability of strains over time and how the continuous application of strain-specific vaccines can modulate the emergence of new strains. Although sampling was limited, this is the first report of subgenotypes 1d and 1e in Chile, both very well supported by Bayesian phylogenetic tree and mPTP analyses. The distance found between these strains and others reported in Chile evidences the continuous need to expand the monitoring in herds and to deepen the analysis of sequences spatially and retrospectively, to define the entry and transmission pathway of the virus, as well as its behavior in terms of mutation and dominance. These results, together with another random sampling, should be considered in the design of new vaccines that will contribute to the successful implementation of viral control and eradication programs in the area.

### Supplementary Information

**Additional file1: Fig. S1.** Significant discrete genetic units or potentials quasispecies variants based on mPTP analysis. Values behind the nodes indicate posterior probability values. Units are indicated in bold letters. The accession numbers are the same as in Figure 1.

**Additional file 2: Table S1.** Accession number and origin of best matches strains compared by blast analysis to the samples in study.

## Data Availability

All data and materials are available for review if necessary.

## Abbreviations

BDV: Border disease virus
BLAST: Basic local alignment search tool
BMCMC: Bayesian Markov chain Monte Carlo
BVDV: Bovine viral diarrhea virus
CSFV: Classical swine fever virus
mPTP: Multi-rate Poisson Tree Process
NCBI: National Center for Biotechnology Information
PCR: Polymerase chain reaction
PI: Persistently infected
SDT: Sequence demarcation tool
UTR: Untranslated region
