# Supplementary material for: First report of bovine viral diarrhea virus subgenotypes 1d and 1e in southern Chile
Source: Virol J. 2023 Sep 7;20:205. doi: 10.1186/s12985-023-02170-4 (PMC10486069; doi:10.1186/s12985-023-02170-4)
Supplement: Supplementary file 2 — Additional file 2: Table S1. Accession number and origin of best matches strains compared by blast analysis to the samples in study. [file 12985_2023_2170_MOESM2_ESM.docx]

**Table S1.** Accession number and origin of best matches strains compared by blast analysis to the samples in study.

| **GenBank Accession number of the samples** | **Subgenotype** | **Identity with best match (%)** | **GenBank accession number of best match** | **Origin** |
| --- | --- | --- | --- | --- |
| OL860949 | 1e | 100 | OP966764 | Brazil |
|  |  | 99,59 | MW655627 | Switzerland |
|  |  | 99,17 | MG434588 | Italy |
| OL860950 | 1b | 97,91 | MG434583 | Italy |
|  |  | 97,91 | LT900977 | United Kingdom |
| OL860951 | 1d | 97,84 | MT036103 | Brazil |
|  |  | 97,41 | KT951841 | China |
| OL860952 | 1e | 100 | OP966764 | Brazil |
|  |  | 100 | MW655627 | Switzerland |
| OL860953 | 1b | 99,17 | MN248507 | Argentina |
|  |  | 99,17 | LT901732 | United Kingdom |
| OL860954 | 1b | 99,59 | MN248507 | Argentina |
|  |  | 99,17 | LT901732 | United Kingdom |
| OL860955 | 1e | 100 | OP966764 | Brazil |
|  |  | 99,59 | MW655627 | Switzerland |
|  |  | 99,18 | MG434588 | Italy |
| OL860956 | 1e | 100 | OP966764 | Brazil |
|  |  | 99,59 | MW655627 | Switzerland |
|  |  | 99,17 | MG434588 | Italy |
| OL860957 | 1e | 99,59 | OP966764 | Brazil |
|  |  | 99,18 | MW655627 | Switzerland |
|  |  | 98,77 | MG434588 | Italy |
| OL860958 | 1e | 99,59 | OP966764 | Brazil |
|  |  | 99,17 | MW655627 | Switzerland |
|  |  | 98,76 | MG434588 | Italy |
| OL860959 | 1e | 100 | OP966764 | Brazil |
|  |  | 99,59 | MW655627 | Switzerland |
|  |  | 99,17 | MG434588 | Italy |
| OL860960 | 1e | 100 | MH902656 | Switzerland |
|  |  | 100 | LT903272 | United Kingdom |
|  |  | 100 | OP966764 | Brazil |
