# Supplementary material for: First report of bovine viral diarrhea virus subgenotypes 1d and 1e in southern Chile
Source: Virol J. 2023 Sep 7;20:205. doi: 10.1186/s12985-023-02170-4 (PMC10486069; doi:10.1186/s12985-023-02170-4)
Supplement: Supplementary file 1 — Additional file1: Fig. S1. Significant discrete genetic units or potentials quasispecies variants based on mPTP analysis. Values behind the nodes indicate posterior probability values. Units are indicated in bold letters. The accession numbers are the same as in Figure 1. [file 12985_2023_2170_MOESM1_ESM.pdf]

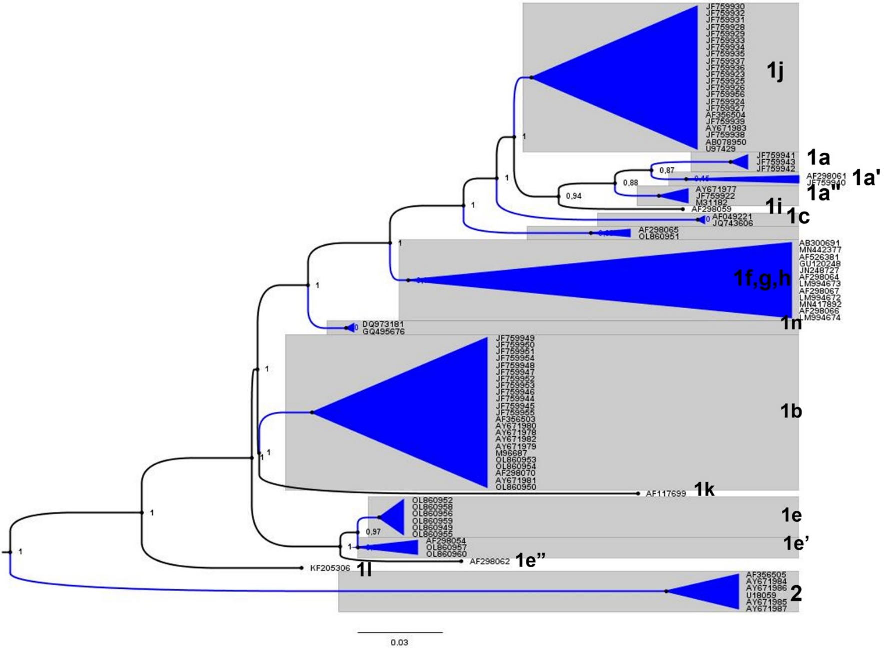

JF759930
JF759932
JF759931
JF759928
JF759929
JF759933
JF759934
JF759935
JF759937
JF759936
JF759923
JF759925
JF759926
JF759956
JF759924
JF759927
AF356504
JF759939
AY671983
JF759938
AB078950
U97429
1j
JF759941
JF759943
JF759942
1a
AF298061
JF759940
1a'
AY671977
JF759922
M31182
1a"
AF298059
1i
AF049221
JQ743606
1c
AF298065
OL860951
AB300691
MN442377
AF526381
GU120248
JN248727
AF298064
LM994673
AF298067
LM994672
MN417892
AF298066
LM994674
1f,g,h
DQ973181
GQ495676
1n
JF759949
JF759950
JF759951
JF759954
JF759948
JF759947
JF759952
JF759953
JF759946
JF759944
JF759945
JF759955
AF356503
AY671980
AY671978
AY671982
AY671979
M96687
OL860953
OL860954
AF298070
AY671981
OL860950
1b
AF117699
1k
OL860952
OL860958
OL860956
OL860959
OL860949
OL860955
1e
AF298054
OL860957
OL860960
1e'
AF298062
1e"
KF205306
1l
AF356505
AY671984
AY671986
U18059
AY671985
AY671987
2
0,87
0,88
0,94
0,97
0.03
